# Albumin-derived perfluorocarbon-based artificial oxygen carriers can avoid hypoxic tissue damage in massive hemodilution

**DOI:** 10.1038/s41598-020-68701-z

**Published:** 2020-07-20

**Authors:** Anna Wrobeln, Johannes Jägers, Theresa Quinting, Timm Schreiber, Michael Kirsch, Joachim Fandrey, Katja B. Ferenz

**Affiliations:** 1 Institute of Physiology, University Hospital Essen, University of Duisburg-Essen, Hufelandstraße 55, 45122 Essen, Germany; 2 Institute of Physiological Chemistry, University Hospital Essen, University of Duisburg-Essen, Hufelandstraße 55, 45122 Essen, Germany; 3 CeNIDE (Center for Nanointegration Duisburg-Essen) University of Duisburg-Essen, Carl-Benz-Strasse 199, 47057 Duisburg, Germany

**Keywords:** Physiology, Respiration, Therapeutics, Drug development, Preclinical research, Translational research

## Abstract

Artificial blood for clinical use is not yet available therefore, we previously developed artificial oxygen carriers (capsules) and showed their functionality in vitro and biocompatibility in vivo. Herein, we assessed the functionality of the capsules in vivo in a normovolemic hemodilution rat-model. We stepwise exchanged the blood of male Wistar-rats with medium either in the presence of capsules (treatment) or in their absence (control). We investigated tissue hypoxia thoroughly through online biomonitoring, determination of enzyme activity and pancreatic hormones in plasma, histochemical and immunohistochemical staining of small intestine, heart, liver and spleen as well as in situ hybridization of kidneys. After hemodilution, treated animals show higher arterial blood pressure and have a stable body temperature. Additionally, they show a more stable pH, a higher oxygen partial pressure (pO_2_), and a lower carbon dioxide partial pressure (pCO_2_). Interestingly, blood-glucose-levels drop severely in treated animals, presumably due to glucose consumption. Creatine kinase values in these animals are increased and isoenzyme analysis indicates the spleen as origin. Moreover, the small intestine of treated animals show reduced hypoxic injury compared to controls and the kidneys have reduced expression of the hypoxia-inducible erythropoietin mRNA. In conclusion, our capsules can prevent hypoxic tissue damage. The results provide a proof of concept for capsules as adequate erythrocyte substitute.

## Introduction

The need to develop synthetic products for blood replacement has become more and more important during the last decades. On the one hand, the demographic change leads to increasing requirements for blood, and on the other hand, simultaneously the willingness for blood donation declines^1^. Blood with its various characteristics fulfils many functions, recent developments of synthetic blood substitutes focus on its most important function: the transport of physiological gases, in particular of oxygen. Additional key requirements for artificial blood substitutes are optimal size, sterility, biocompatibility, and the ability to be stored without loss of functionality. Moreover, artificial oxygen carriers (AOCs) should have a long intravascular circulation time and universal usage independent of the blood group characteristics^2,3^. There are two main approaches to realize the transport of essential gases. The first is the use of the physiological oxygen carrier hemoglobin (hemoglobin-based artificial oxygen carriers, HBOCs); a concept realized e.g. in the prominent product hemopure, that gained pharmaceutical approval in South Africa and Mexico. Further HBOCs (e.g. sanguinate) are currently tested in clinical trials^4^. So far, most of the products showed severe side effects so that the manufacturing had to be stopped^5^. The second is the work with synthetic materials like perfluorocarbons [perfluorocarbon-based artificial oxygen carriers (PFOCs)]. Depending on the actual partial pressure, PFOCs are able to solve gases physically^6^. In contrast to HBOCs, PFOCs are applicable for therapy of decompression sickness, smoke/carbon monoxide poisoning and recently in tumour therapy^7–10^. Because of their general chemical inertness, they remain functional even in the presence of carbon monoxide and hydrogen cyanide^6^. Their inertness also prevents enzymatic degradation and avoids toxic intermediates, which might harm organs. However, the physical properties cause a distinct hydro- and lipophobicity, which requires the development of a blood compatible form for intravenous application^11,12^.

While HBOCs need a certain minimum size to prevent local nitric oxide scavenging from the endothelium in order to avoid hypertension, PFOCs tend to enlarge due to Oswald ripening and flocculation^3^.

We successfully synthesized nanoscaled PFOCs with a perfluorodecalin (PFD) core surrounded by a biocompatible albumin shell (capsules)^13^. Previous studies successfully describe the in vivo biocompatibility in a rat-top-load model and the functionality in an ex vivo isolated rat heart model^11,14,15^. Herein, we used a rat model of massive hemodilution, where we exchanged about 95% of the blood volume with either capsules in a plasma-like solution (treatment) or the plasma-like solution without capsules (control) and posthumously surveyed hypoxia sensitive organs such as the small intestine and kidney. In the context of AOC development, normovolemic hemodilution of the rat is a well-established model to test their functionality^16–19^. Our study is aimed at serving as a “proof of concept” that capsules are a life-saving erythrocyte substitute, thereby avoiding the onset of tissue hypoxia even at critically low hematocrit.

## Results

### Bioparameters

#### Survival

**Figure 1:**
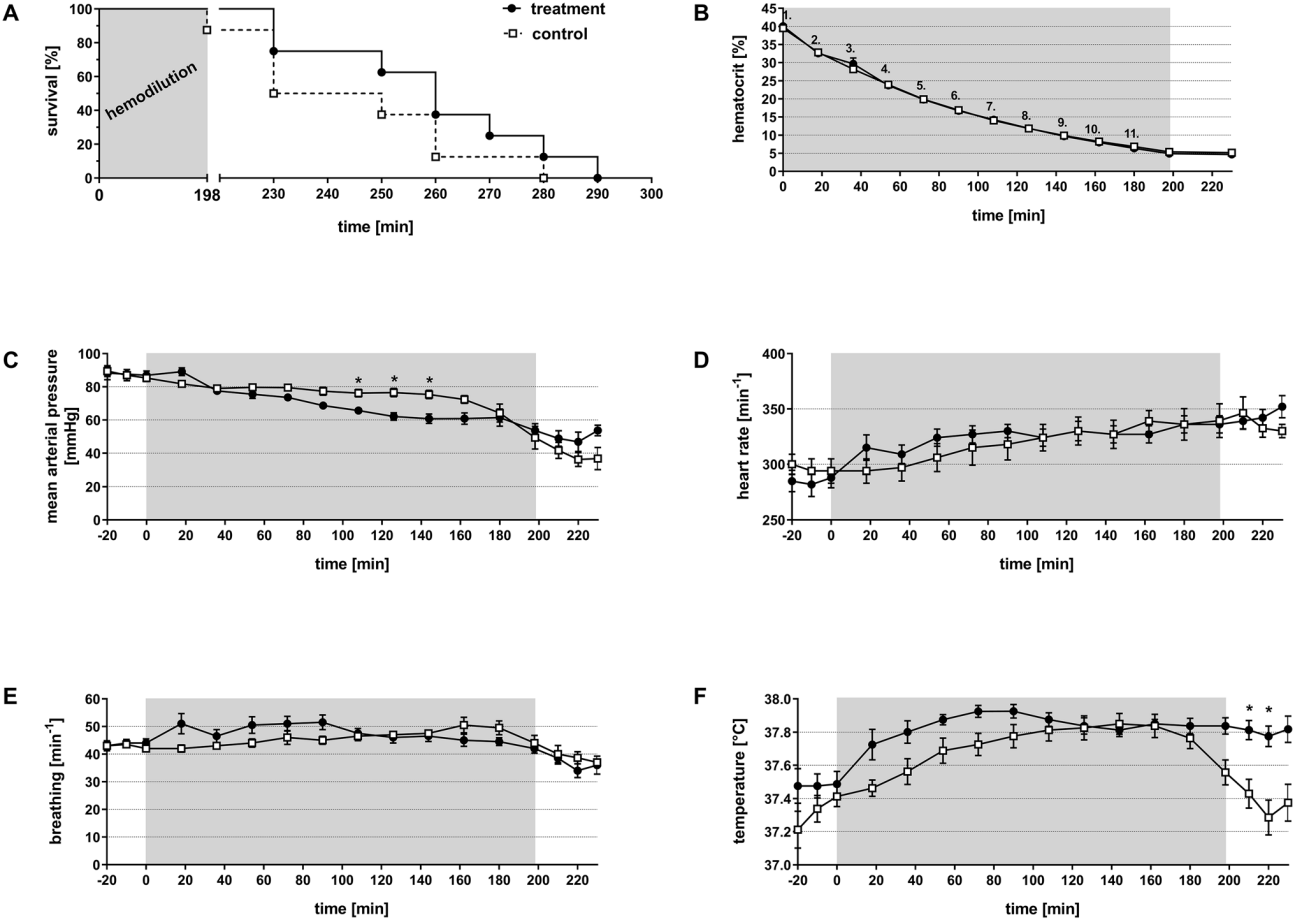
Effect of normovolemic hemodilution on survival (**A**), hematocrit (**B**) mean arterial pressure (**C**), heartrate (**D**), breathing (**E**) and temperature (**F**). During hemodilution (grey background), animals were diluted using 5% human serum albumin [HSA (control)] or 12 vol% capsules (treatment) to a hematocrit of 5%. The Kaplan–Meier plot shows an increased survival of the treatment group compared to the control animals (**A**). The plots show the mean ± SEM of n = 8 animals per group. Asterisk indicates significance with p < 0.05 compared to the controls*.*

After establishing the hematocrit of 5%, we closely monitored all animals until death. All animals survived the hemodilution period. Figure 1A shows that during the subsequent observation period the first animal of the control group died in minute 198 (at the end of the dilution phase/start of the observation period), which is 32 min earlier than the first two animals of the treatment group, that died in minute 230. At the same time (230 min), two additional animals of the control group died as well. Subsequently, one animal of each group died in minute 250, followed by two animals of each group in minute 260. Ultimately, the last animal of the control group died in minute 280 of the experiment. At this time, three animals of the treatment group were still alive. Two of them died in minute 270 and 280, and the last one died in minute 290. Overall, the treated animals survived longer compared to the controls. Whilst 50% of the control animals had died after 230 min, 75% of the treatment animals were still alive. In the following sections all parameters are statistically compared until minute 230 of the experiment (200 min hemodilution + 30 min post-dilution observation period).

As shown in Fig. 1B, we equally reduced hematocrit levels stepwise in both groups until all animals stayed constantly at a haematocrit ~ 5%. Therefore, the actual hematocrit per se can unlikely serve as the explanation for the tendency of different survival times between the groups and the following parametric results.

#### Systemic parameters

During the whole experiment we registered, mean arterial pressure (MAP), heart rate, breathing and temperature continuously (Fig. 1C–F). At the beginning of the experiment, both groups started with a MAP of 90 mmHg, which decreased continuously in both groups (Fig. 1C). The animals of the control group showed a MAP of 76.2 ± 2 mmHg in minute 108, whereas the animals of the treatment group reached a significantly lower value of 65.7 ± 2 mmHg (p < 0.05). This significant difference remained until minute 142. However, following that, the animals of the treatment group could stabilize their MAP at about 50 mmHg while in the control group the MAP declined continuously. After the period of hemodilution, the MAP in animals of the treatment group fell to 53.7 ± 3 mmHg (in minute 230) compared to a MAP of 36.8 ± 5 mmHg in the animals of the control group.

The heart rate was virtually identical in the two groups (Fig. 1D). Animals of the control group started with a heart rate of 300 ± 9 min^−1^. A similar heart rate with 285 ± 10 min^−1^ was recorded for the treatment group. The heart rate of both groups increased during the experiment, to a final heart rate of 330 ± 4 min^−1^ (control) and 352 ± 9 min^−1^ (treatment). Both groups showed a continuously similar heart rate during the experiment.

In both groups, breathing of animals was largely constant in the same range (Fig. 1E). In detail, the animals of both groups started with a frequency of 43.0 min^−1^ which remained stable until the end of the hemodilution (minute 198; control group: 44.0 ± 3 min^−1^; treatment group: 42.0 ± 2 min^−1^). After the period of hemodilution, breathing rate decreased in both groups, ending up in 36.0 ± 1 min^−1^ in minute 230.

Body temperature of the animals (Fig. 1F) was 37.2 ± 0.1 °C for the control group and 37.5 ± 0.1 °C for the treatment group at the beginning of the experiment. During the experiment, the temperature of both groups increased to 37.8 ± 0.1 °C in the control animals and 37.9 ± 0.0 °C in the capsule-treated animals in minute 180. 18 min before the end of the hemodilution period (from minute 180), body temperature of the control animals started to drop, finally reaching 37.4 ± 0.1 °C in minute 230. Body temperature of the animals of the treatment group remained constant.

### Acid base status and metabolic parameters

**Figure 2:**
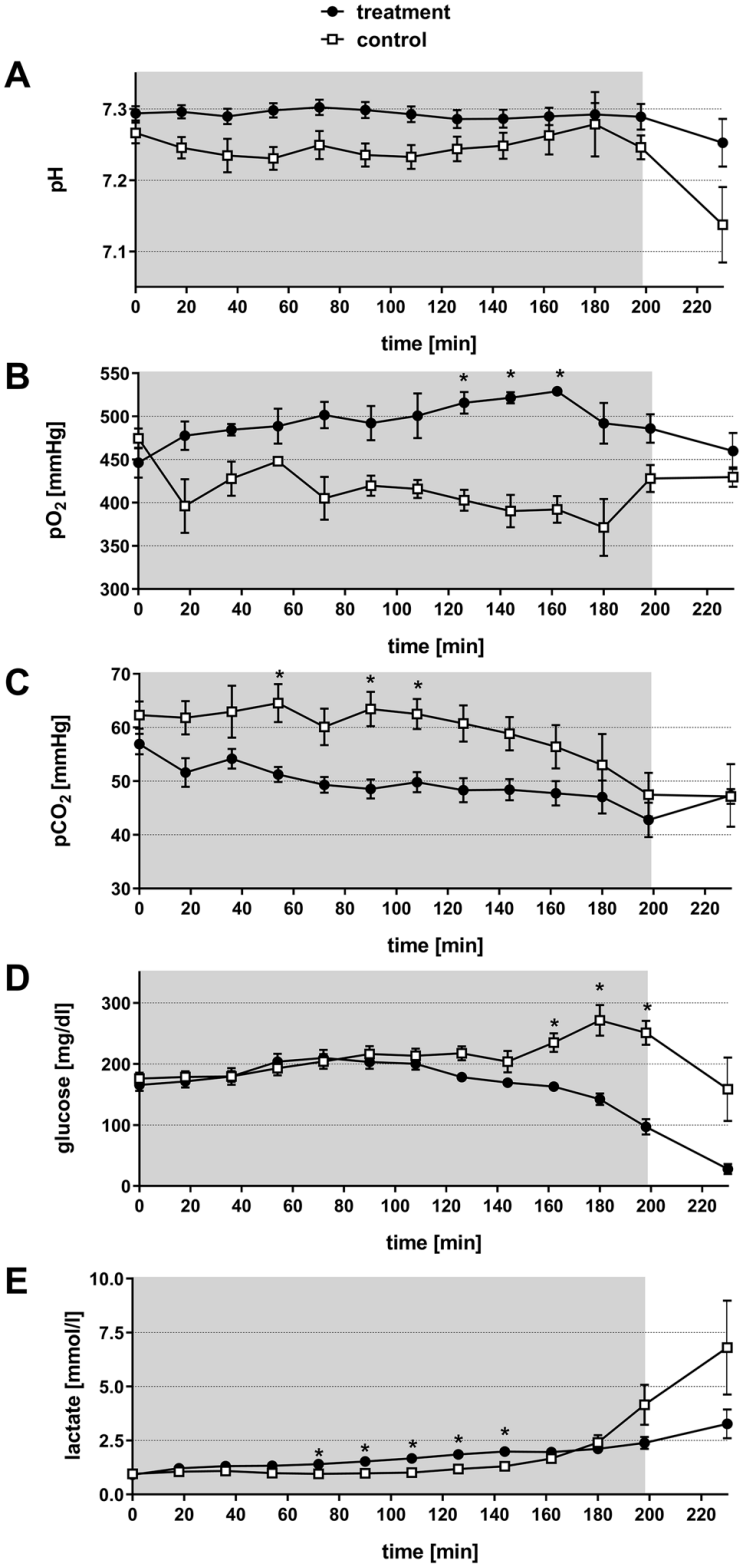
Effect of normovolemic hemodilution on blood pH-value (**A**), pO_2_ (**B**), pCO_2_ (**C**), glucose (**D**) and lactate (**E**). During hemodilution (grey background), animals were diluted using 5% HSA (control) or 12 vol% capsules (treatment) to a hematocrit of 5%. The plots show the mean ± SEM of n = 8 animals per group. Asterisk indicates significance with p < 0.05 compared to the controls.

The data on acid base status and metabolic parameters revealed significant differences during the hemodilution period (Fig. 2).

The blood pH of the animals in both groups remained stable at a similar level from the beginning of the experiment until the end of the dilution period (Fig. 2A). Within this experimental period, the pH of the control animals varied between pH 7.23 and pH 7.28 and the pH of the capsule-treated animals between pH 7.21 to pH 7.30. Post-dilution pH in the animals of the control group, dropped to a final acidotic value of pH 7.14 ± 0.1 (minute 230) while the pH value of the treatment group remained stable (pH 7.25 ± 0.1).

During the whole experiment, the arterial pO_2_ of the animals in the control group was below the pO_2_ level of the capsule-treated animals. At several time points the difference reached significant levels (Fig. 2B). Immediately after the start of the dilution a clear difference of 81 mmHg between the two groups was noticed (minute 18: control: 396.1 ± 31 mmHg, treatment: 477.6 ± 15 mmHg). This difference increased throughout the dilution period and reached significance in minute 126, 144 and 162. At minute 162, the maximum difference between the two groups, with 137 mmHg, was observed (control: 392.1 ± 15 mmHg, treatment: 529.1 ± 4 mmHg).

The arterial pCO_2_ from animals of the control group was consistently higher than the pCO_2_ of treatment group (Fig. 2C). In addition, a difference of 10 mmHg between both groups was noticed immediately after the start of the dilution (minute 18) (control: 61.8 ± 3 mmHg, treatment: 51.6 ± 3 mmHg). This difference remained until the end of the dilution period with significantly different values at minute 90. At the end of the post-dilution period (minute 230) the pCO_2_ values of the two groups aligned (control: 47.2 ± 1 mmHg, treatment: 47.3 ± 5 mmHg).

Blood glucose concentration (Fig. 2D) were similar for both groups at the beginning of the experiment (control: 176.1 ± 10 mg/dl, treatment: 165.6 ± 10 mg/dl). In animals of both groups, blood glucose concentration moderately increased until minute 108 (control: 213.2 ± 12 mg/dl, treatment: 203.1 ± 11 mg/dl). Subsequently, blood glucose concentration of the control animals increased from minute 144 to 198 and was significantly higher than in the animals of the treatment group (p < 0.05). At minute 180 control animals reached the maximum glucose concentration of 271.4 ± 25 mg/dl, which decreased to 158.5 ± 52 mg/dl at minute 230 but always remained above the level of the capsule-treated animals (minute 230: 27.8 ± 7 mg/dl).

Blood lactate concentration of the animals (Fig. 2E) was around 2 mmol/l in both groups until minute 160, significant differences occurred between the two groups in minute 90, 108 and 126. From minute 180, the blood lactate values of the control animals strongly increased to 6.8 ± 1.5 mmol/l in minute 230. In comparison, blood lactate levels of capsule-treated animals only marginally increased to a final concentration of 3.3 ± 0.6 mmol/l.

### Organ damage

**Figure 3:**
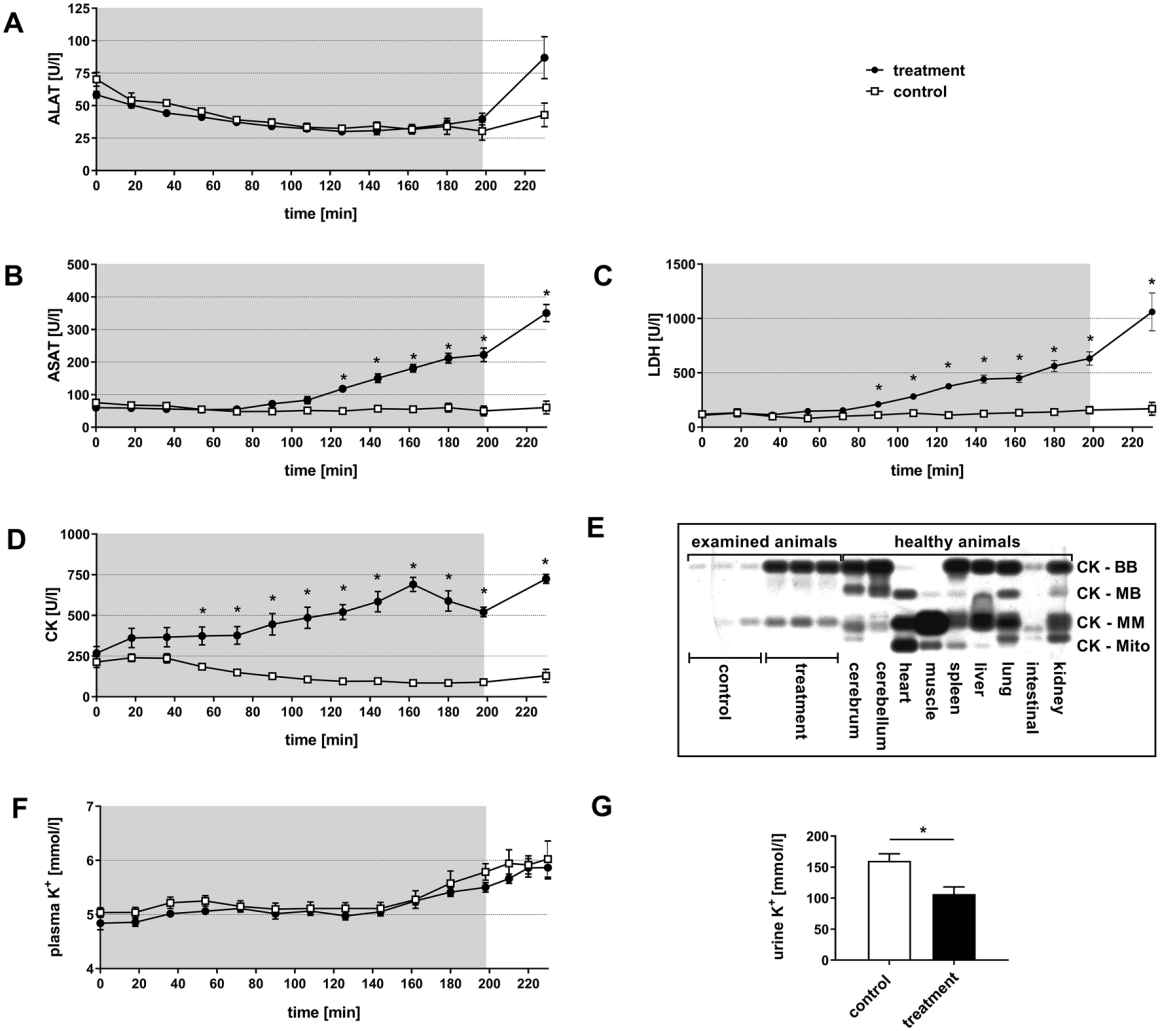
Effect of normovolemic hemodilution on ALAT (**A**), ASAT (**B**), LDH (**C**), CK (**D**), CK isoenzymes (**E**) and K+ (**F**) in plasma in addition to K+ in urine (**G**). During hemodilution (grey background), animals were diluted using 5% HSA (control) or 12 vol% capsules (treatment) to a hematocrit of 5%. The plots (A-D, F, G) show the mean ± SEM of n = 8 animals per group. Asterisk indicates significance with p < 0.05 compared to the controls. CK-isoenzyme determination (**E**) in respectively three plasma samples per group of minute 162 (examined animals). Control animals showed slight bands of CK-BB and CK-MM, whereas the treatment group showed increased bands of CK-BB and CK-MM. Comparison of CK-isoenzyme pattern of the treatment group with the organ-specific pattern of organ homogenates of cerebrum, cerebellum, heart, muscle, spleen, liver, lung, intestinal and kidney of healthy untreated animals matched best with the spleen-specific pattern of CK-isoenzymes treatment bands with organ homogenates.

The evaluation of intracellular enzymes in the plasma (Fig. 3) as indicator for organ damages showed significant differences between the two groups. Alanine aminotransferase (ALAT), aspartate aminotransferase (ASAT), creatine kinase (CK) and lactate dehydrogenase (LDH) remained within the normal range in animals of the control group over the whole experiment. In contrast, in animals of the treatment group ALAT increased during the post-intervention period from 58.4 ± 3 U/l at the beginning of the experiment to 86.9 ± 14 U/l at minute 230, but was not significantly elevated compared to the animals of the control group (Fig. 3A). The aminotransferase ASAT (Fig. 3B) showed a significant increase in activity from 60.3 ± 4 U/l at the beginning of the experiment to 118.1 ± 9 U/l (at minute 126) and further increased to 350.5 ± 23 U/l (at minute 230).

Similarly, LDH continuously increased in capsule-treated animals from minute 90 onwards and was significantly different from the control group (control: 112 ± 23 U/l, treatment: 210 ± 28 U/l; (Fig. 3C), finally (minute 230) reaching a maximum of 1,060 ± 150 U/l in the blood plasma.

CK levels (Fig. 3D) started to rise immediately after the start of hemodilution in the treatment group. This increase reached a significantly enhanced level in minute 54, compared to the control group. At minute 230, the CK activity of capsule-treated animals was 724.8 ± 24 U/l compared to the control group with 128.8 ± 28 U/l.

CK-isoenzyme determination (Fig. 3E) of plasma CK showed an increase of CK-BB and CK-MM bands in the treatment group compared to controls. Comparison of the CK-isoenzyme pattern of the treatment group with the organ specific patterns of organ homogenates of cerebrum, cerebellum, heart, muscle, spleen, liver, lung, intestine and kidney matched closest with the spleen-specific pattern of CK-isoenzymes.

Determination of plasma K^+^ levels (Fig. 3F) showed an increase in both experimental groups, without significant differences. In the control animals, plasma K^+^ increased from 5.04 ± 0.09 mmol/l at the beginning of the experiment to 6.03 ± 0.34 mmol/l at minute 230. The treatment animals showed an increase from 4.84 ± 0.12 mmol/l at the start of the experiment to 5.87 ± 0.21 mmol/l at minute 230.

Urine K^+^-concentration was 160.1 ± 11.3 mmol/l in the control animals and significantly lower with 106.4 ± 11.7 mmol/l in the treatment group (Fig. 3G).

### Histology

#### Spleen

**Figure 4:**
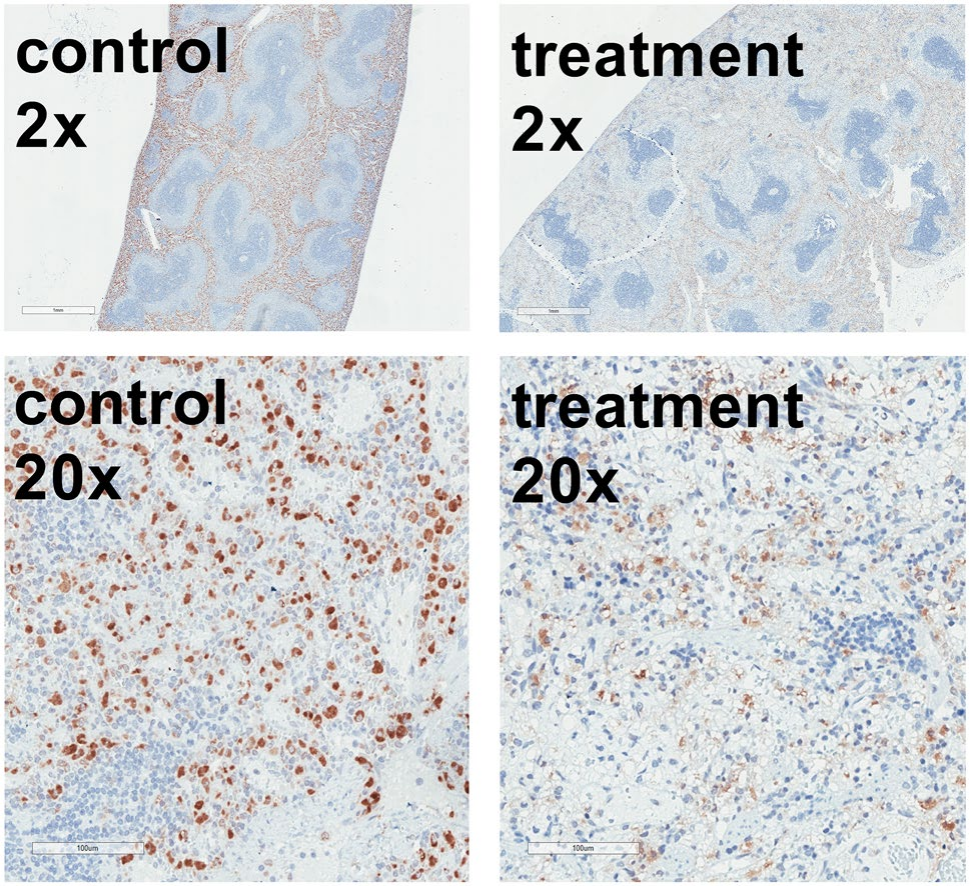
Macrophage staining of the spleen. Immunohistochemical staining with CD68 clone ED1 antibody of spleen tissue of animals diluted using 5% HSA (control) or 12 vol% capsules (treatment). Hematoxylin (blue) co-staining was used for orientation. The brown colour represents the macrophages. Control animals showed unaffected macrophages in contrast to foamy vacuolized macrophages in the treatment group. Staining was performed with n = 8 spleens in each group. Treatment spleens demonstrated lost tissue structure within the red pulpa with foamy, swollen macrophages. The white pulpa was not affected. The control spleens were intact.

Histological evaluation of the spleen demonstrated lost tissue structure within the red pulpa in the treatment group, whereas the white pulpa was not affected. In comparison, the spleens of the control animals were unaffected. Further immunohistological staining for CD 68 clone ED1 revealed foamy, swollen macrophages in the red pulpa of treatment animals (Fig. 4).

#### Liver

**Figure 5:**
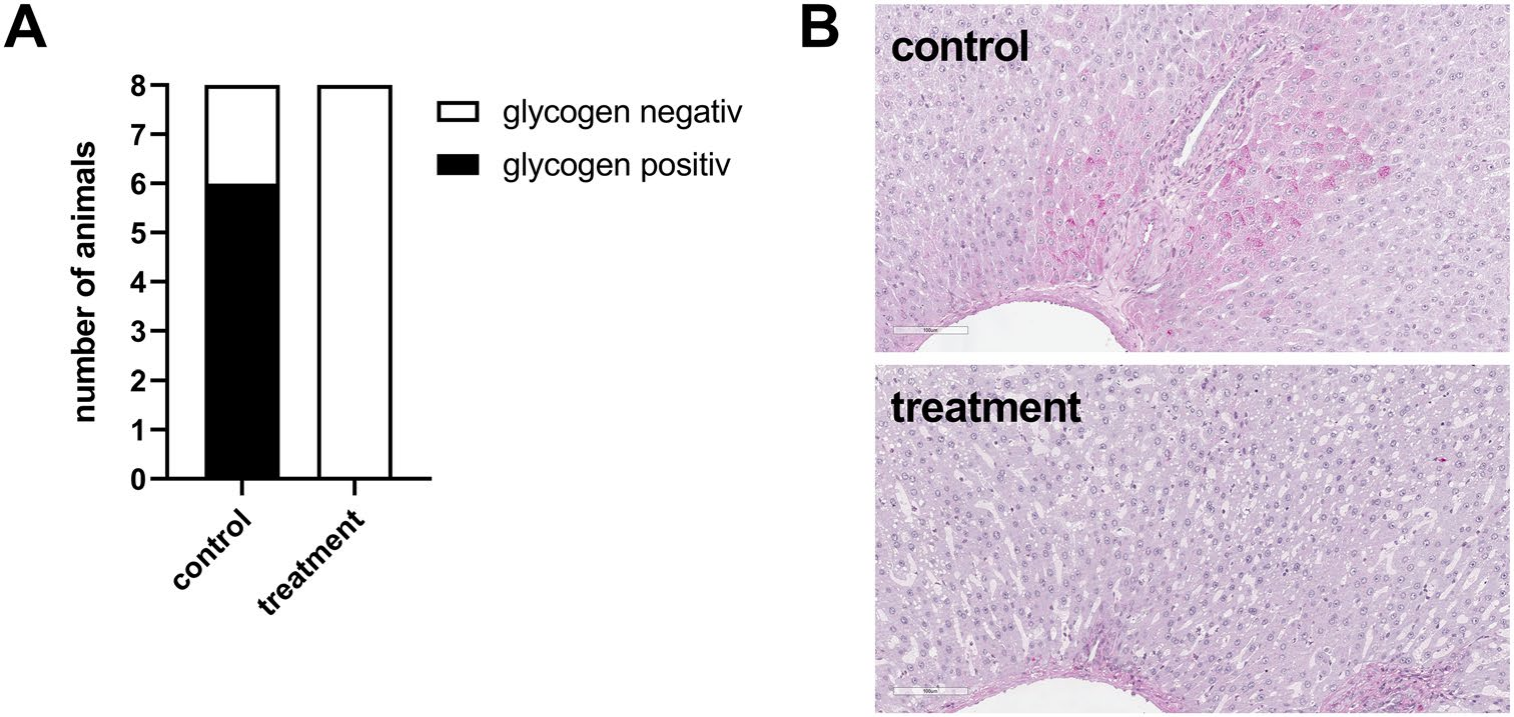
Qualitative determination of glycogen appearance in liver. PAS/hematoxylin staining in liver tissue of animals diluted using 5% HSA (control) or 12 vol% capsules (treatment). In the control group, 6 of 8 livers were glycogen positive, whereas no liver of treatment animals showed presence of glycogen (**A**). Representative examples of liver staining are shown in (**B**).

Qualitative analysis of periodic acid-Schiff reaction staining (PAS) for glycogen revealed a complete depletion of the glycogen reserve in animals of the treatment group. Spots of stained glycogen were completely missing in the livers of these animals. Only livers of the control group heterogeneously stained for glycogen as shown in Fig. 5A and in the exemplary pictures in Fig. 5B.

#### Intestine

**Figure 6:**
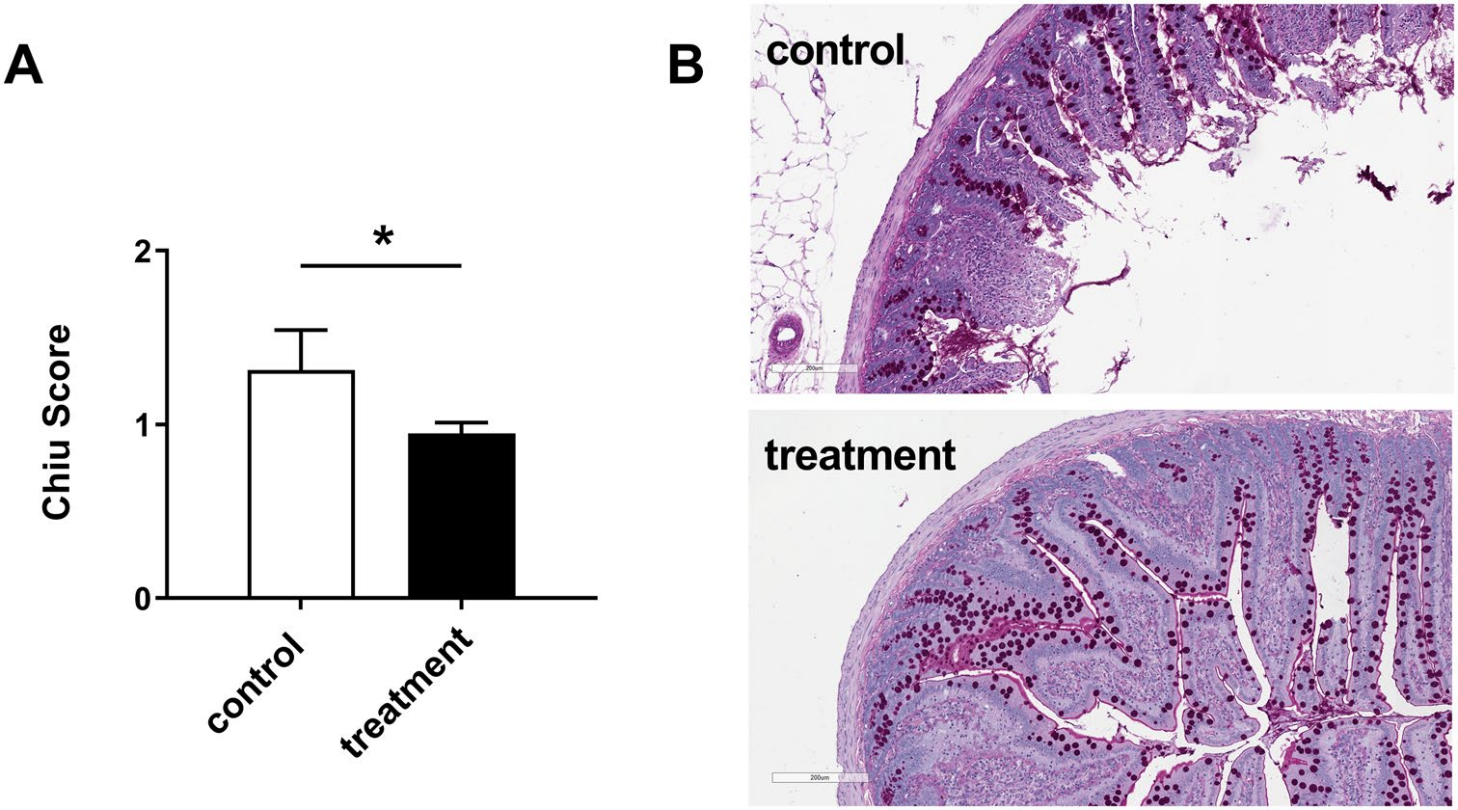
Quantification of ischemic injury in the small intestine. PAS/hematoxylin staining in small intestine tissue of animals diluted using 5% HSA (control) or 12 vol% capsules (treatment). (**A**) The occurring ischemic injury in the small intestine of rats in the small intestine of rats in the treatment group was significantly reduced compared to the control group. (**B**) Representative examples of the intestine staining.

The scoring of the ischemic damage of the small intestine was significantly elevated in the control group, where the *villi* showed Gruenhagen spaces and even denudation. In contrast, the small intestines from rats of the treatment group showed mostly intact *villi*, sometimes with small Gruenhagen spaces. Intestine of the control group reached more Chiu score points for tissue damage up to a score of 4, whereas small intestine of the treatment group rarely had more than 2 score points (Fig. 6A). Figure 6B shows representative examples of the observed tissue damage in both groups.

### Assessment of pancreatic hormones in plasma

**Figure 7:**
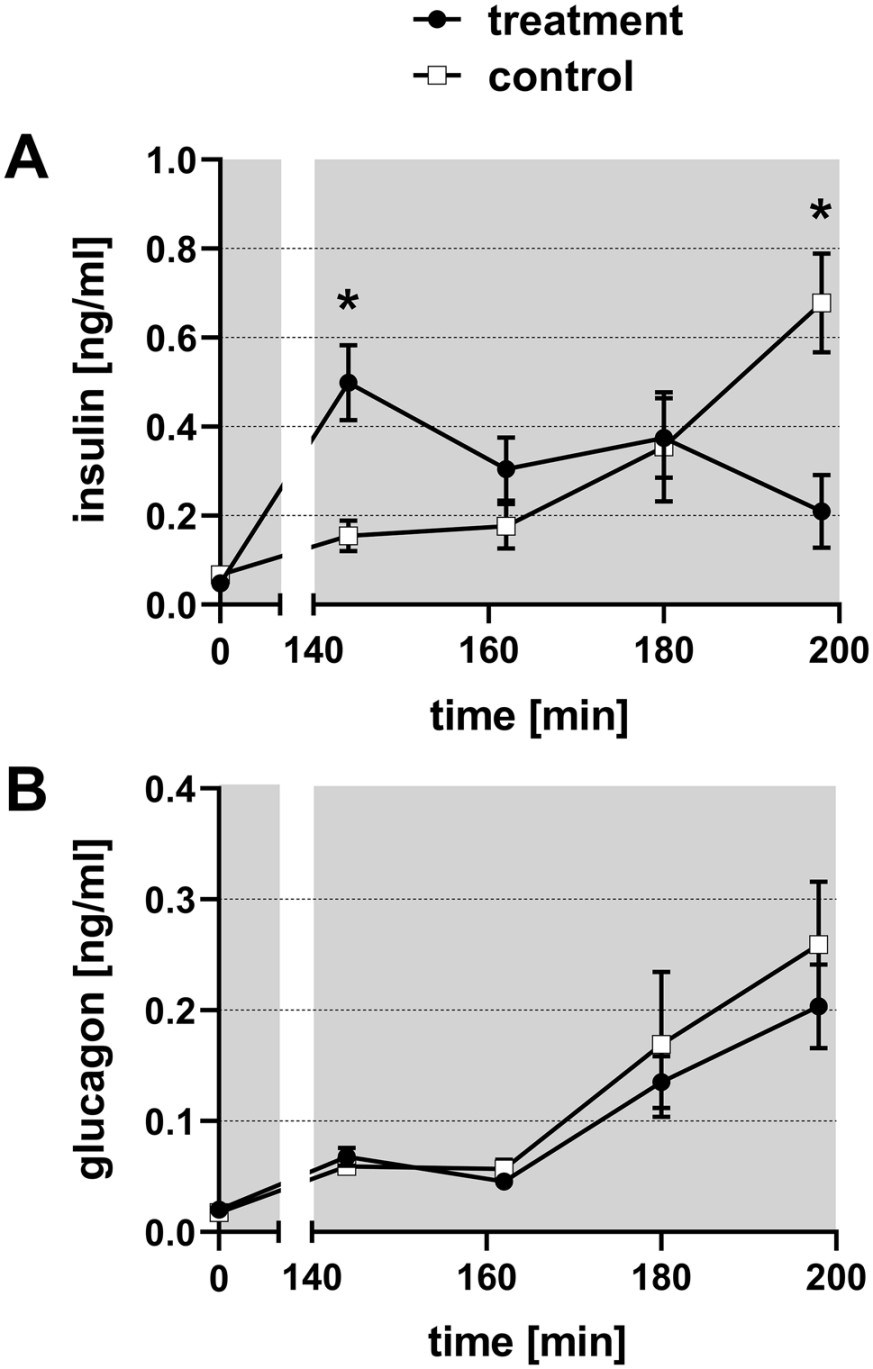
Effect of normovolemic hemodilution on plasma insulin (**A**) and plasma glucagon (**B**). During hemodilution (grey background), animals were diluted using 5% HSA (control) or 12 vol% capsules (treatment) to a hematocrit of 5%. The plots show the mean ± SEM of n = 8 animals per group. Asterisk indicates significance with p < 0.05 compared to the controls.

Baseline value for insulin (0.05 ± 0.01 ng/ml) and glucagon (0.02 ± 0.0 ng/ml) blood concentration were obtained directly after placement of the catheter. According to the measured glucose concentrations, plasma samples from minute 144, 162, 180 and 198 were analysed for insulin and glucagon. Figure 7A demonstrates the decrease of the insulin concentration from 0.50 ± 0.09 ng/ml (min 144) to 0.21 ± 0.08 ng/ml (min 198) in the treatment group, while plasma glucose concentration constantly fell as shown in Fig. 2D. In contrast, plasma insulin concentration rose at the same time to 0.68 ± 0.11 ng/ml in the control group. Glucagon levels (Fig. 7B) in both groups increased from minute 144 from 0.06 ng/ml to 0.20 ± 0.04 ng/ml (min 198) in the treatment group and to 0.26 ± 0.06 ng/ml (min 198) in the control group.

### Quantification of renal EPO-mRNA

**Figure 8:**
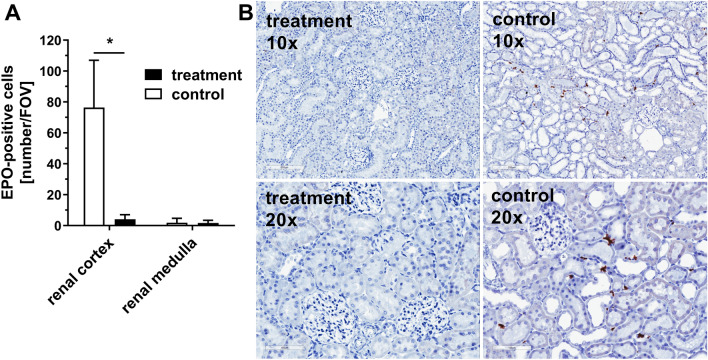
Quantification of renal EPO-mRNA. Animals were diluted using 5% HSA (control) or 12 vol% capsules (treatment) to a hematocrit of 5%. Kidneys were used for EPO-mRNA in situ hybridization. (**A**) Number of EPO-positive cells per field of view (FOV) in the in the renal cortex or the renal medulla of the kidney after hemodilution. (**B**) Representative pictures of the EPO-mRNA in situ hybridization used for quantitative analysis. The brown staining demonstrates the EPO-mRNA. In situ hybridization was performed with n = 8 kidneys in each group. For determination of EPO-positive cells 10 randomly chosen FOV per renal cortex or 5 randomly chosen FOV per medulla were counted.

Figure 8A demonstrates the number of erythropoietin (EPO)-mRNA positive cells in the observed fields of view (FOV). Cells in the renal medulla of both groups showed only very few EPO-mRNA positive cells. However, comparison of the renal cortex of both groups indicated that the amount of EPO-mRNA positive cells was significantly increased in the animals of the control group (76.4 ± 31 cells/FOV) compared to the kidneys of animals from the treatment group (4.1 ± 3 cells/FOV). Figure 8B shows representative examples of kidneys after EPO-mRNA in situ hybridization. Control animals showed specific EPO-mRNA expression (Fig. 8B, left pictures) whereas kidneys of treatment animals were essentially free from staining for EPO-mRNA (Fig. 8B, right pictures).

## Discussion

In vitro experiments, perfusion of isolated *Langendorff* hearts as well as in vivo toxicities studies showed promising results for the applicability of albumin-derived perfluorocarbon-based capsules as blood substitutes and dispelled concerns on capsule aggregation in vivo^13,15^. Still, physiological in vivo “proof of concept” was missing. In the context of AOC development, normovolemic hemodilution of the rat is a well-established model to test functionality of a novel AOC^16–19^. Consequently, we used the model of massive hemodilution in the rat to determine the physiological functionality of our capsules. In detail, rats were diluted from their physiological hematocrit of about 45% below the critical hematocrit. The critical organ hematocrit is organ specific, but about 10% for the whole rat and indicates the hematocrit that is necessary for oxygenation of the most relevant organs and thus for survival of the animal^20^. With a target hematocrit of 5% we have purposely gone below this critical hematocrit to make the effects of a deficient oxygen supply obviously visible in the control animals. Similar approaches were performed in other studies evaluating novel AOCs^21–23^.

In the present study any differences in the dilution protocol between the two groups were meticulously eliminated, as all animals (regardless of group affiliation) showed the same hematocrit levels at each dilution step (Fig. 1B). In general, the experiment showed no significant differences in survival of rats after massive hemodilution (Fig. 1A). However, the MAP of the treatment animals temporally decreased significantly compared to the control animals, but was terminally stabilized in the treatment animals (Fig. 1C) resulting in a longer survival (Fig. 1A). Neither for heart rate (Fig. 1D) nor for breathing rate (Fig. 1E) was a significant difference detected. A possible reason for that could be the loss of heart rate- and breathing rate-response due to the adjustment of anaesthesia with isoflurane^24,25^. Interestingly, the animals of the treatment group stabilized their body core temperature after the period of hemodilution, whereas the animals of the control group showed a decrease in temperature (Fig. 1F). Gellhorn et al*.* demonstrated that body core temperature decreases due to a lack of oxygen^26^. In this work a deficiency of O_2_ correlated with an increase of CO_2_. The increased enrichment of the vasodilatory molecule CO_2_ results in a dilatation of the peripheral blood vessels, which was observed similarly by Sakai et al*.*^27^. Moreover, this dilatation ultimately leads to a loss of body temperature through body surface. In line with Gellhorn’s results the animals of the control group showed a lower pO_2_ (Fig. 2B) and a higher pCO_2_ (Fig. 2C), compared to the animals of the treatment group. Presumable, the loss of body core temperature, as characterized by Gellhorn et al*.*, is attributable to the deficient oxygen supply and the simultaneous increase of pCO_2_ in the control animals. In contrast, the treatment animals showed a higher pO_2_ and lower pCO_2_ and could thereby maintain their body core temperature.

During hemodilution the animals of the treatment group sustained a stable pH (Fig. 2A), which allows to conclude a positive influence on the acid base status by the capsules. Nevertheless, animals of the treatment group showed a decrease in plasma glucose concentration although the solution used for hemodilution contained physiological plasma glucose concentrations (10 mM) of the rat (Fig. 2D). To exclude a hormonal influence on blood glucose-level due to dysregulated pancreatic function, we analysed pancreatic hormones (Fig. 7). The animals of the control group showed an increase in both hormones, which indicates a dysregulation of the pancreas in the control group and not as expected in the capsule group. This loss of physiological function may be due to hypoxic conditions. In contrast, animals of the treatment group showed a decrease of insulin and an increase of glucagon, which represents the physiological reaction of the pancreas to a decreasing plasma glucose level. Unexpectedly, treatment animals showed an increase of insulin although the plasma glucose already started to decline. We therefore shifted our attention towards the liver as glucose storage and supplier. In general, the liver is able to compensate a decline of plasma glucose levels due to gluconeogenesis and glycogenolysis. In case of loss of function, the liver is not able to activate the glycogen reserves or synthesize glucose to stabilize the blood glucose level anymore. Because a parallel increase of the plasma-enzyme activities of ALAT and ASAT (liver damage parameters, Fig. 3A, B) takes place just prior to death of the animals (minute 230), a general dysfunction of the liver was excluded as a cause for the glucose decline. However, alterations of the liver through PFCs were already described in the literature. Obviously, the reason is storage of PFCs in the hepatic Kupffer-cells^28–32^. Like in the actual investigation, Lutz et al*.* showed an increase in plasma levels of ALAT and ASAT in their studies. In comparison to the present study, they determined the plasma enzyme activities over eight days. Importantly, this increase was only transient and normalized to physiological levels within 24 h. Thus, they concluded that no ultimate damage of the liver occurred but a rather transient functional impairment^32^. Because of a different study design, we do not have any information on plasma levels of ASAT and ALAT after 24 h. Nevertheless, our data support the hypothesis that the liver was not damaged but rather impaired in the present study, as depleted glycogen-stores in every liver of the treatment animals were likely the reason for plasma glucose decrease (Fig. 5). This is a hint for an intact liver as glycogenolysis obviously took place in these animals. The treatment animals were able to use all their metabolic reserves to survive, because of the ability of oxygen for metabolic processes. The reason for dying of these animals seems to be a kind of starvation. At the end of the experiment, the rats had consumed all their reserves to a point where they failed to stabilize their metabolism via both glycogenolysis and gluconeogenesis.

In contrast, control animals seemed to be insufficient to mobilize their metabolic reserves for surviving, which might be due to a hypoxic status and could be the reason for dying. In any case, in the control animals the progression of plasma glucose level is more clear (Fig. 2D). During the hemodilution period, when more and more organs reach their critical hematocrit, animals showed a typical stress reaction (increase in plasma glucose). With progressive oxygen shortage (during the post-hemodilution phase), remaining glucose was used for anaerobic glycolysis, reflected in increasing lactate levels (Fig. 2E). The fact that plasma lactate did not increase in capsule-diluted animals supports the hypothesis of sufficient oxygenation of those animals.

The treatment animals showed an increase of CK and LDH plasma levels (Fig. 3C,D). As mentioned before, several studies indicate that Kupffer-cells of the liver or macrophages of the spleen preliminary absorb perfluorocarbon- containing particles^28,30–32^. Comparison of organ-specific isoenzyme pattern of healthy rats with plasma patterns from animals of this study (Fig. 3E) indicates that increased plasma CK might be caused by structural changes in the spleen. A histological and immunohistological evaluation of the spleen demonstrated loosened structure in the red pulpa in all animals of the treatment group (Fig. 4). The macrophages were enlarged and vacuolized, confirming the literature. CK is part of the stress fibers, which are located in the splenic sinus endothelium^33^. The enlarged macrophages require more space, which could be the reason for injured stress fibers that probably released CK in the surrounding area and lead to the increased plasma CK. Previous studies showed similar alterations of the spleen after administration of identical capsules^13^.

To further support the hypothesis, that capsules are capable of avoiding hypoxic tissue damage in severely anaemic animals, the organs most sensitive to hypoxic damage were analysed; namely the kidney with its oxygen-dependent proximal tubules and the intestine with its juxta-positioned mucosa and submucosa physiologically approaching critical oxygen levels^34–36^. To evaluate the oxygenation status of the kidney, renal peritubular fibroblasts that express the mRNA of hypoxia inducible factor (HIF)-target gene EPO were used as a sensitive detection system^37–39^. HIF, in its role as oxygen sensing protein of the cell, upregulates endogenous adaption mechanisms such as the EPO pathway that principally leads to erythropoiesis, in order to prevent hypoxic injury^40,41^. An early activity sign of this pathway is EPO-mRNA upregulation^42^. The treatment animals showed a significant lower number of renal EPO-mRNA expressing cells (Fig. 8). Furthermore, as expected EPO-expressing cells appeared in the renal cortex but not in the renal medulla (Fig. 8A)^39^. These data further support the notion that on the cellular level capsule-diluted animals were less hypoxic during the experiment compared to the control group that showed an increased number of EPO expressing cells.

Beside the findings in the kidney, the observation of the small intestine further confirmed these results. Due to the microbial environment of the lumen, this tissue already physiologically exhibits low concentrations of oxygen entailing that hypoxic injury of the intestine is well characterized^35,36^. That limits the tolerance towards oxygen depletion caused by any reason, such as arterial occlusion, heart failure or hemodilution. The intestine of the control group showed massive degradation of the *villi* in terms of loss of epithelium, degradation of the submucosa and hemorrhages (Fig. 6). In contrast, the intestine of the animals of the treatment group only showed marginal, if any, occurrence of Gruenhagen spaces, which are the first sign of a beginning damage (Fig. 6). This data strongly supports the hypothesis that capsules are able to avoid hypoxia in treatment animals, as oxygen supply was sufficient to prevent hypoxic conditions in the two most sensitive organs, the kidney and the small intestine. This might allow the assumption, that all other organs in the body were supplied with enough oxygen, too. Probably the animals of the treatment group died due to the lack of nutrition and carbohydrates, whereas the animals of the control group died because of severe global hypoxia that lead to multi modal organ dysfunction and ultimately to the death of the animals.

## Conclusion

The model of normovolemic hemodilution was chosen as the adequate way in order to test the ability to run a whole organism on albumin-derived perfluorocarbon-based artificial oxygen carriers instead of pure blood. The capsules successfully supplied the organisms with sufficient amounts of oxygen despite severe loss of erythrocytes. All systemic parameters were stabilized using capsules, whereas blood gases, namely the measured pO_2_ and pCO_2_ were ameliorated by the capsules gas-transporting capacity. The further analyses of pancreatic hormone status, renal oxygen-sensitive cells, liver function, status of intestine and spleen tissue illustrate that the beneficial aspects of the capsules widely out do the presumable transient damage of the spleen that leads to a rise of CK in plasma. Importantly, oxygen supplied by capsules reached the cells and thus prevented hypoxia even on the cellular level.

The next step will be the investigation of albumin-derived perfluorocarbon-based artificial oxygen carriers, referring to clinical reality, against red blood cell concentrates in hemodilution and hemorrhagic shock models. The performance in long-term studies will proof the transient effects of the capsule toward the spleen.

## Limitations of the study

- This study was designed as a proof of concept study without any clinical interventions during hemodilution and observation period. This of course impaired total survival time of the animals of both groups. Common transfusion triggers followed by many clinicians are hematocrit levels of 24% or lower, so that hematocrit levels of 5% are rarely met in the clinic. In addition patients would not be left untreated but stabilized with assisted ventilation and pharmaceuticals. We resigned from such interventions and chose this artificial scenario which has been commonly used in the HBOC/PFOC field to prove, that our capsules supply a whole organism with oxygen in the absence of most of the endogenous erythrocytes.
- Plasma contains many important ingredients besides oxygen carriers, free albumin molecules, hormones, electrolytes (Na^+^, K^+^, Mg^2+^, Ca^2+^, Cl^−^) and amino acids. However, our carrier solution (again according to cited similar studies by other groups investigating their AOCs) consisted of only water, albumin, sodium chloride and 10 mM glucose (the physiological glucose plasma concentration in rats) which is similar to other AOC carrier solutions and exclusively investigated the functionality of the capsules. For clinical applications further ingredients are necessary, such as buffer components, amino acids and hormones.
- Following the principles of 3 R (reduction, refinement, replacement) which is generally acknowledged by all animal researchers, we reduced the animals within our study and did not include a second control group (hemodilution with washed erythrocytes).
- In contrast to the hematocrit the fluorocrit at different time points (and thus the actual concentration of our capsules) was not determined in this study which prevents calculating the actual total concentration of capsules in the blood. A hypothetical estimation is difficult and imprecise as continuous capturing of capsules within the spleen and removal of some capsules with every dilution step until the end of hemodilution need to be taken into consideration. Without touching the system (without continuous hemodilution) the circulatory half-life of the product was measured to be 158 min^13^.

## Methods

### Capsule synthesis

As previously described 5 ml of 5% human serum albumin (HSA) (Baxter (Unterschleissheim, Germany, 5% HSA, 0.75% NaCl, 0.11% sodium-*N*-acetyltryptophanoate, 0.07% sodiumcaprylate) and 1 ml Perfluorodecalin (PFD; Fluorochem Chemicals, Derbyshire, UK) were combined in a reaction tube with a total capacity of 15 ml^13^. The temperature of the reaction tube was controlled using an ice bath and the mixture was sonicated for 90 s using a sonotrode with a tip diameter of 3 mm associated with a UP 400S ultrasonic processor (Hielscher, Teltow, Germany). For sonication, the tip of the sonotrode was placed at the PFD–water interface. At a power of 400 W, ultrasound with amplitudes of 210 mm and a frequency of 24 kHz was generated.

After synthesis, the capsule volume fraction (vol%) was determined using microhematocrit glass capillary tubes (d = 1.15 mm, Brand, Wertheim, Germany) and a centrifuge (Universal 320R, Hettich, Tuttlingen, Germany) with a hematocrit rotor. Analog to the hematocrit determination of blood we used 16,060 g for 15 min at 4 °C for determination of volume fraction. Based on the determined volume fraction capsules were diluted to 12 vol%. We used the capsules straight after synthesis and adaption of volume fraction for animal experiments. The capsules’ main properties are displayed in Table 1.

**Table 1:** Main properties of capsules.

Capsules		
Mean diameter	479 ± 36 nm	
Polydispersity index	0.56 ± 0.05	
Circulatory in vivo half-life	158 min	

	Capsules	Whole blood (Hct 45%)
O_2_ capacity (pO_2_ = 713 mmHg)	3.11 ml O_2_/dl	~ 20 ml O_2_/dl
O_2_ release (pO_2 100% O2 aspiration_ → pO_2 venous_)	3.10 ml O_2_/dl	~ 4 ml O_2_/dl

Previously determined mean diameter ± SD, polydispersity index ± SD, circulatory in vivo half-life as well as calculated O_2_-capacity and -release of capsules and whole blood^13^.

### Animals

Male Wistar rats (Rattus norvegicus, 430–460 g) were obtained from the central animal unit of the Essen university hospital. Animals were kept under standardized temperature conditions (22 ± 1 °C), humidity (55 ± 5%) and 12/12-h light/dark cycles with free access to food (ssniff-Spezialdiaeten, Soest, Germany) and water. All animals received humane care according to the standards of Annex III of the directive 2010/63/EU of the European Parliament and of the Council of 22 September 2010 on the protection of animals used for scientific purposes^43^. The experimental protocol was approved by the North Rhine-Westphalia state office for Nature, Environment and Consumer Protection (Landesamt fuer Natur, Umwelt und Verbraucherschutz Nordrhein-Westfalen), Germany under the reference number 84-02.04.2016.A118, based on the local animal protection act.

### Anaesthesia, analgesia, and surgical procedure

As previously described, rats were anesthetized with isoflurane (2.0% in 100% medical O_2_ at 4.0 l/min for induction, 1.5–2.0% isoflurane in 100% medical O_2_ at 1.0 l/min throughout the experiment) through face masks connected to a vaporizer (Isofluran Vet. Med. Vapor; Draeger, Luebeck, Germany)^13^. For analgesia, they received ketamine subcutaneously (50 mg/kg body weight) into the right chest wall. After local lidocaine administration (5 mg/kg body weight subcutaneously), a median skin-deep inguinal incision of about 2 cm was made along the right groin and a Portex catheter (0.58 mm ID, 0.96 mm OD; Smith medical, Grasbrunn, Germany) was placed within the right femoral artery and the right femoral vein. Each catheter was fixed with a surgical suture.

### Hemodilution

Hemodilution was performed as described previously, with some modifications^44^. After an initial stabilization period, 3 ml blood was taken from the femoral arterial catheter at a rate of 1 ml/min by hand until reaching a final hematocrit of 5 ± 1%. The removed blood was replaced simultaneously (to withdrawal) over the venous catheter via a syringe pump (KDS Legato 100, Cole Parmer, Illinois, USA) and used for further analysis (see “Blood parameters”). After every withdrawal, a period of 15 min followed to stabilize the circulation of the animal. A 5% HSA solution with 10 mM glucose (control) or a 5% HSA solution with 10 mM glucose containing 12 vol% capsules (treatment) were applied as exchanging solutions. After finishing dilution, animals were monitored until death. Each group consisted of n = 8 animals.

### Biomonitoring

Systemic and vital parameters were monitored every 10 min through the whole experiment. The femoral arterial catheter was connected to a pressure transducer for continuous measurement of systolic and diastolic blood pressure, the mean arterial pressure (MAP) was calculated out of these datasets. To keep the catheter functional, Ringer Saline (Fresenius Kabi AG, Germany) was infused at a rate of 3 ml/h. The pressure peaks of the continuous blood pressure measurement were used to determine the heart rate. The breathing rate was calculated based on the number of ventilation movements in 15 s. A rectal sensor was used to measure the body core temperature (temperature), which was conserved with the help of a thermostat-controlled operating table and covering the animal with aluminium foil.

### Blood parameters

Blood samples were analysed for blood gas analyses and enzyme activities at different time points. Therefore, 0.5 ml blood were either diverted from the 3 ml obtained during a hemodilution step anyway (during the hemodilution period for time points minute 0, 18, 36, 54, 72, 90, 108, 126, 144, 162, 180, see “Hemodilution”) or additionally removed from the femoral artery catheter (during the post-intervention period for time points minute 198, 230, 260 and final, depending on the animal survival time) using a 2 ml syringe containing 80 international units (IU) electrolyte-balanced heparin (Pico50, Radiometer Medical, Brønshøj, Denmark). Blood was centrifuged at 4500*g* for 10 min at room temperature to obtain plasma. During the experiment blood gas analysis of the arterial blood pH, oxygen and carbon dioxide partial pressures (pO_2_, pCO_2_), concentration of glucose and lactate was performed with a blood gas analyser (ABL 715, Radiometer, Copenhagen, Denmark). The collected plasma was used to measure plasma enzyme activities of aspartate aminotransferase (ASAT) and alanine aminotransferase (ALAT) as markers for hepatocyte injury, lactate dehydrogenase (LDH) as a general marker of cell injury as well as the creatine kinase (CK) as a marker for muscle cell injury. Enzyme activities were determined using a fully automated clinical chemistry analyzer (Respons 920, DiaSys, Holzheim, Germany). Plasma concentrations of insulin and glucagon were determined in blood samples obtained in minute 0, 144, 162, 180 and 198 using a commercially available rat-specific immune assay (Ultra sensitive Rat Insulin ELISA Kit and Rat Glucagon ELISA Kit, Chrystal Chem, Downers Grove, USA). The assays were performed according to the manufactures instructions.

### Urine parameter

At the end of the experiment, after death of the animal, urine was collected by puncturing the bladder. After dilution (1:10) with urine diluent (Easy Lite, Medica, Bedford, Netherlands), urinary K+-concentration was determined by the help of a blood gas analyser (ABL 715, Radiometer, Copenhagen, Denmark).

### CK-isoenzymes

Plasma samples with the highest CK-activity (minute 162) were chosen to assess CK-isoenzymes using a Hydragel ISO-CK Test Kit (Sebia Labordiagnostische Systeme GmbH, Fulda, Germany). The plasma-CK-isoenzyme-cluster was compared with pooled organ homogenates from different organs (heart, spleen, liver, muscle, lung, kidney, intestine, cerebrum and cerebellum) taken from healthy rats to determine the origin of CK. Organs were obtained from five Wistar rats after euthanazation and kidney removal for another project, in line with the guidelines of the North Rhine-Westphalia state office for Nature, Environment and Consumer Protection (Landesamt fuer Natur, Umwelt und Verbraucherschutz Nordrhein-Westfalen), Germany. In detail, isoenzymes were separated using agarose gel electrophoresis; and afterwards stained due to the manufactures instructions.

### Quantification of EPO-mRNA

RNA in situ hybridization was performed on 3 µm thick formalin-fixed paraffin-embedded sections of rat kidneys using the RNAscope 2.5 HD assay-brown (Advanced Cell Diagnostics) according to the user manuals 322452-USM and 322310-USM using standard conditions. EPO-mRNA was detected by the RNAScope probe Rn-Epo (Cat No. 455901) followed by semi-quantitative scoring, calculating the average count of EPO producing cells per field of view in the kidney cortex and medulla. In situ hybridization was performed with n = 8 kidneys in each group. For determination of EPO-positive cells 10 randomly chosen FOV per renal cortex or 5 randomly chosen FOV per medulla were microscopically analysed and counted [Aperio CS2 (Leica Biosystems), objective: UPlanSApo 20x/0.75 (Olympus)].

### Histology

Tissue sections were fixed in 4% paraformaldehyde (Carl Roth, Karlsruhe, Germany) and embedded in paraffin. 1–4 µm sections were stained according to the following protocols.

#### PAS-staining

Glycogen staining of the liver was performed using the periodic acid-Schiff reaction (PAS). The tissue slices were deparaffinised and rehydrated using xylol followed by a descending alcohol series [2 × 100%, 1 × 96%, 1 × 70%, 1 × aqua destillata (aqua dest.)]. The slices were oxidized in periodic acid (Carl Roth, Karlsruhe, Germany) for 15 min at room temperature, washed in aqua dest. for 5 min and stained for 15 min with Schiff reagent (Merck, Darmstadt, Germany) at room temperature. Counterstaining was performed with 25% hematoxylin for 10 s.

#### Immunostaining

Spleen macrophages were stained as described previously using an antibody against the antigen ED:1 (BIOLOGO, Kronshagen Germany, diluted 1:10,000 in phosphate-buffered saline), the rat homologue of human CD68 which is expressed by the majority of tissue macrophages and weakly by peripheral blood granulocytes^13^.

### Chiu score for quantification of ischemic injury of the small intestine

The small intestine was divided into ten equally sized segments and the fourth segment (numbered from proximal to distal) was chosen to be fixed in 10% formalin (Carl Roth, Karlsruhe, Germany), dehydrated and stored in paraffin. The segment was sliced into 1–2 µm thin slices and PAS-stained with hematoxylin counterstaining. For the analysis, the samples of eight rats per group were randomized and five rings of each segment were evaluated using the Chiu-score for quantification of ischemic injury of the small intestine.

The Chiu-score is an easy and reproducible histological score to evaluate ischemic injury in the small intestine^45^. The scoring points are set from zero to five (0 = no injury, 1 = formation of subepithelial Gruenhagen-spaces at the tip of the villus, 2 = wide subepithelial Gruenhagen-spaces with lifting of the epithelium, 3 = severe lifting of the epithelium and appearance of denuded villus, 4 = denuded villi show exposed lamina propia and capillaries, 5 = severe degradation of the lamina propria, ulceration and hemorrhage).

### Qualitative analysis of the glycogen reserve of the liver

The glycogen reserve of the liver was PAS-stained as described above. The organs of eight rats per group were randomized and the general presence of glycogen was investigated. Doing this, there was no emphasis put on the amount of the glycogen (no quantitative analysis). If spots of glycogen were found, the whole liver counted as “glycogen positive”, if no glycogen was detected, the whole liver counted as “glycogen negative”. The light microscopic analysis was performed at a 20-fold magnification. The whole organ was examined in order to not miss any glycogen spots.

### Statistics

The statistic was analysed using GraphPad prism 8. Data are expressed as mean values ± SD for in vitro experiments and ± SEM for in vivo experiments. Comparisons between groups were analysed by multiple t-test and statistical significance was determined by using the Holm-Sidak post-hoc method considering a p-value < 0.05 to indicate statistical significance. Survival of the animals is shown and compared from the beginning of the hemodilution (minute 0) until the end of the experiment (minute 300). Biomonitoring parameters are shown and compared from the beginning of the experiment (− 20 min) until minute 230 of the experiment. Blood parameters are shown and compared from the beginning of the hemodilution (minute 0) until minute 230 (200 min hemodilution + 30 min post-dilution observation period). This period of 250 min in total was chosen to allow for statistical analysis. At time point minute 198, 100% of the animals were still alive, which reduced down to 50% (control group) and 75% (treatment group) at minute 230. For survival analysis (Kaplan–Meier plot Fig. 1A) all animals were included in the analysis (log-rank test, minute 0–300).

## Supplementary information

Supplementary information

## Data Availability

The datasets generated during and/or analysed during the current study are available from the corresponding author on reasonable request.
