# Supplementary material for: Albumin-derived perfluorocarbon-based artificial oxygen carriers can avoid hypoxic tissue damage in massive hemodilution
Source: Sci Rep. 2020 Jul 20;10:11950. doi: 10.1038/s41598-020-68701-z (PMC7371727; doi:10.1038/s41598-020-68701-z)
Supplement: Supplementary file 1 — Supplementary information [file 41598_2020_68701_MOESM1_ESM.docx]

# Albumin-derived Perfluorocarbon-based Artificial Oxygen Carriers Can Avoid Hypoxic Tissue Damage in Massive Hemodilution

Dr. Anna Wrobeln^1^, M.Sc. Johannes Jägers^1^, M.Sc. Theresa Quinting^1^, Dr. Timm Schreiber^1^, Prof. Michael Kirsch^2^, Prof. Joachim Fandrey^1^, Prof. Katja B. Ferenz^1,3*^

^1^University of Duisburg-Essen, Institute of Physiology, University Hospital Essen,

Hufelandstraße 55, 45122 Essen, Germany

^2^University of Duisburg-Essen, Institute of Physiological Chemistry, University Hospital Essen, Hufelandstraße 55, 45122 Essen, Germany

^3^CeNIDE (Center for Nanointegration Duisburg-Essen) University of Duisburg-Essen, Carl-Benz-Strasse 199, 47057 Duisburg, Germany

*corresponding author
E-mail: katja.ferenz@uk-essen.de

# Supplementary figures


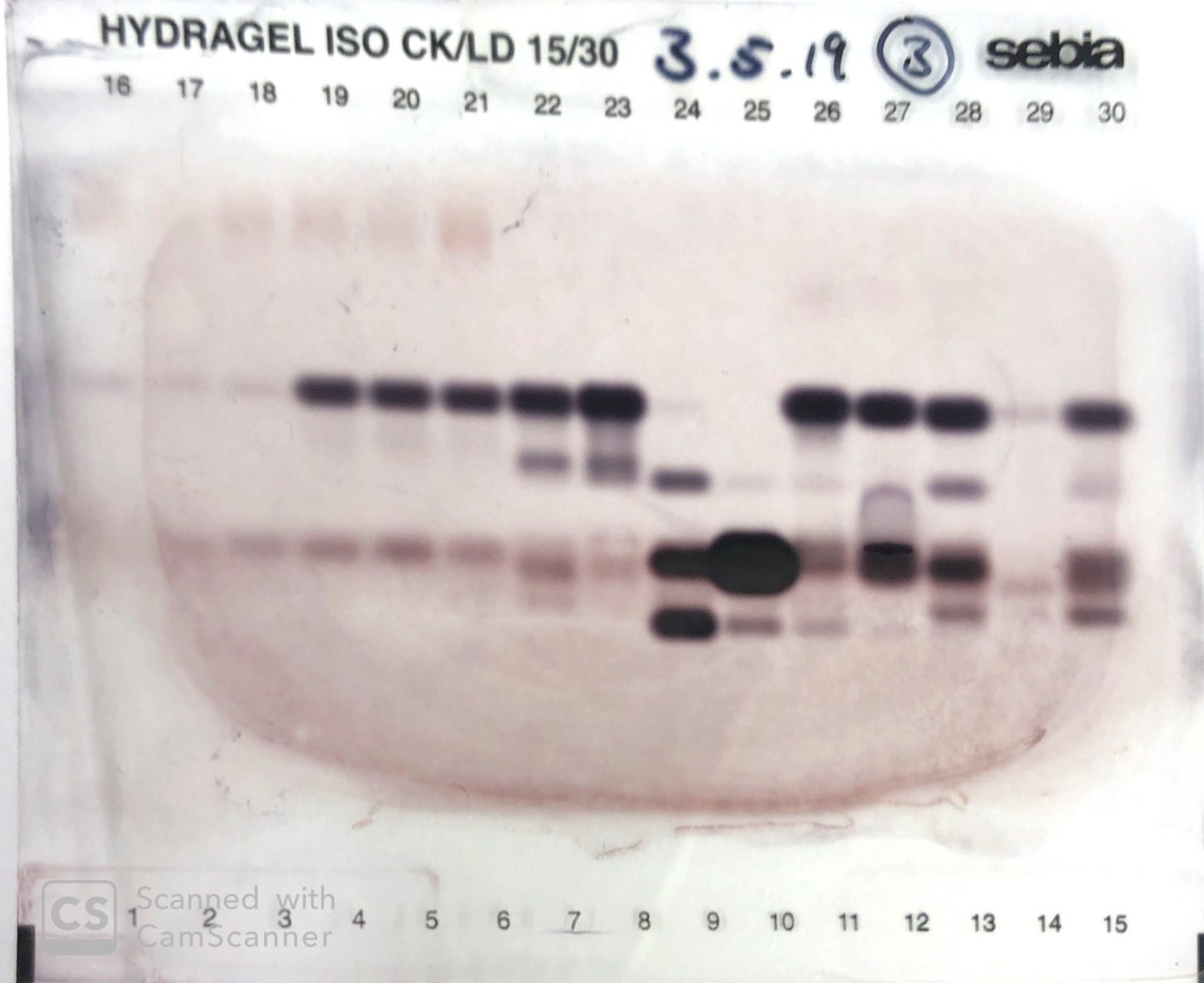


**Supplementary Figure 1: CK-isoenzymes.** Full image of agarose gel from CK-isoenzyme determination presented in figure 3 E.
